# The Use of Inner Retinectomy to Relieve Inner Retinal Foreshortening Causing Retinal Detachment in the Setting of Branch Retinal Vein Occlusion

**DOI:** 10.1155/2020/8853425

**Published:** 2020-06-13

**Authors:** Katsuya Yagisawa, Takayuki Baba, Tomomi Kaiho, Hirotaka Yokouchi, Shuichi Yamamoto

**Affiliations:** Department of Ophthalmology and Visual Science, Chiba University Graduate School of Medicine, Chiba, Japan

## Abstract

A 61-year-old Japanese woman presented with impairment of her left vision due to macular schisis secondary to branch retinal vein occlusion. Her left vision was 20/50, and schisis was observed inferotemporally. She underwent phacoemulsification and aspiration, implantation of the intraocular lens, and removal of the epiretinal membrane and internal limiting membrane. Her visual acuity stabilized ~20/50 for two and a half years after the initial surgery. However, she developed macula-involving retinal detachment, and her visual acuity declined to counting fingers. She underwent pars plana vitrectomy and removal of the residual vitreous cortex together with the inner retina within the area of vein occlusion. After the removal of silicone oil and the addition of an encircling buckle, the retina remained attached and visual acuity improved to 20/60 at one year after the final surgery. The combination of rhegmatogenous and tractional detachment in the area of schisis was suspected, and vitrectomy with inner retinectomy was effective.

## 1. Introduction

Branch retinal vein occlusion (BRVO) is known to occur in 0.5 to 1.2 per 100 persons [1]. The pathogenesis of BRVO is mainly compression of veins at A-V crossings, degenerative changes of vessel walls of the vein, and hypercoagulability. Hypertension is the most common risk factor, and hyperlipidemia and diabetes follow. Age is also associated with the occurrence of BRVO, and the prevalence is 1.57 per 1000 in ages 40-49, 4.58 per 1000 in 50-59, 11.11 per 1000 in 60-69, and 12.76 per 1000 in 70-79 [2].

Reported complications of BRVO include macular edema [3], retinal neovascularization [4], vitreous hemorrhage [5], retinal break [6], and retinal detachment [7]. Serous retinal detachment is observed in 63% of major BRVO cases [8] and is more common than rhegmatogenous retinal detachment (RRD), which was reported as 1.3% [6] to 3% [9, 10]. On the other hand, retinal schisis is a rare condition, and there is no systematic study reporting the prevalence and treatment for the pathology.

In this case report, we presented a case with macula-involving retinal schisis secondary to BRVO that was treated with pars plana vitrectomy (PPV). In the postoperative period, the case developed RRD that was finally reattached by PPV with inner retinectomy.

## 2. Case Report

**Figure 1:**
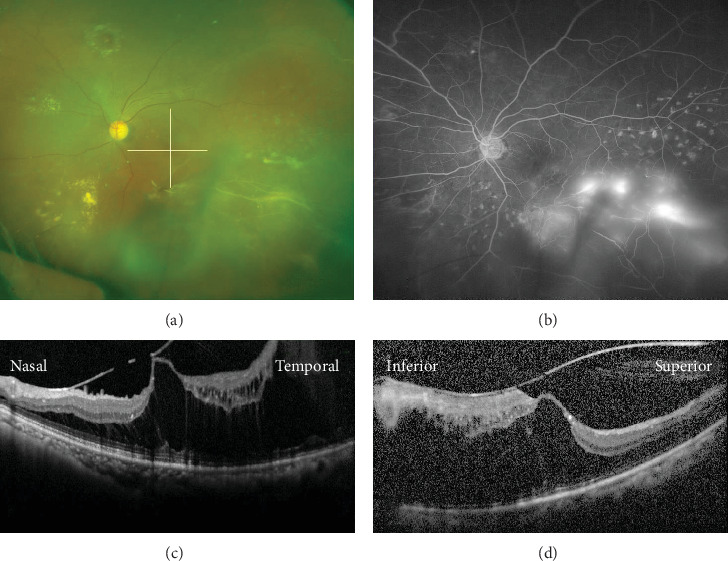
Preoperative images of a 61-year-old Japanese woman with macula-involving retinal schisis secondary to branch retinal vein occlusion (BRVO). (a) An ultrawidefield fundus photograph showing BRVO with white vessels at the inferior temporal retina. The visual acuity in the left eye was 20/50. The lines indicate the location of OCT scans presented in (c) and (d). (b) Fluorescein angiography showing retinal neovascularization in the area of BRVO. (c) A horizontal optical coherence tomography (OCT) image through the macula showing macular traction and extensive retinal schisis. (d) A vertical OCT image through the macula showing broad adhesion of the vitreous and retinal schisis.

**Figure 2:**
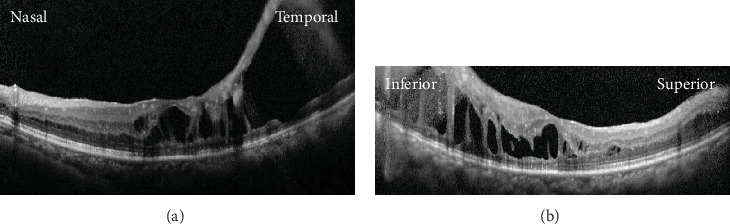
Postoperative images at two months after phacovitrectomy and membrane peeling for retinal schisis. (a) A horizontal optical coherence tomography (OCT) image showing the decreased height of retinal schisis. The schisis cavity is still wide in the temporal retina. (b) A vertical OCT image showing decreased but persistent retinal schisis.

**Figure 3:**
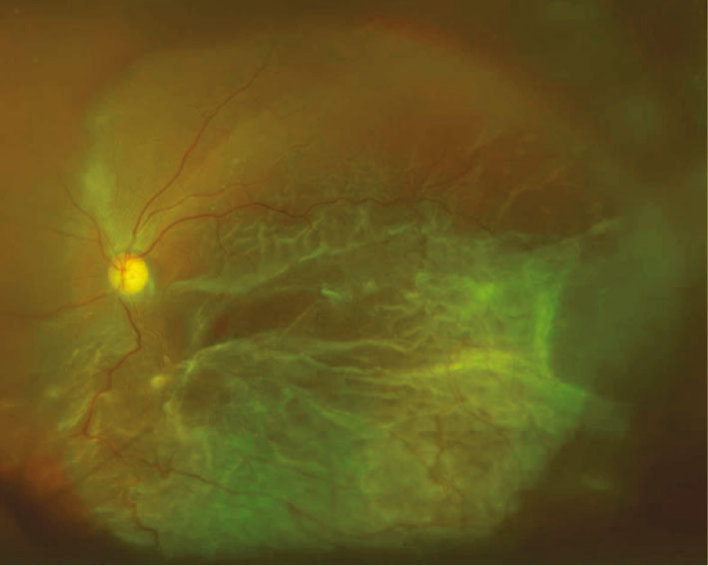
A preoperative image before the second vitrectomy. An ultrawidefield fundus photograph showing the retinal detachment involving the macula at two and a half years after the initial surgery. The visual acuity in the left eye was counting fingers.

**Figure 4:**
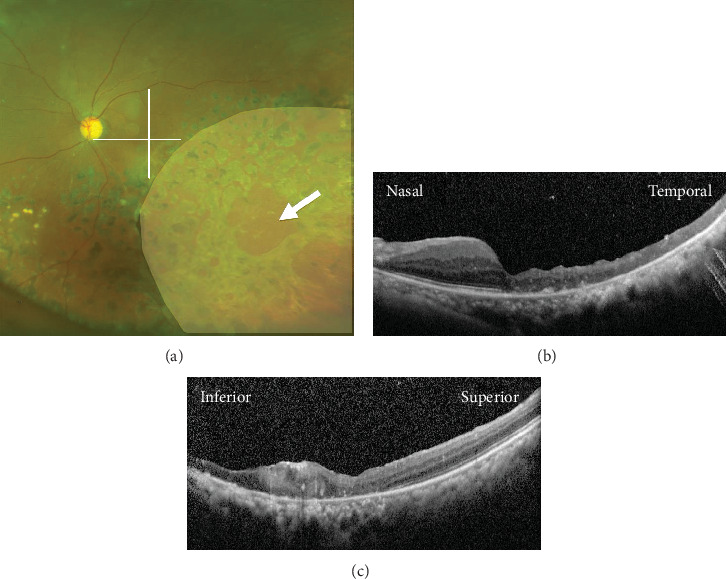
Postoperative images at one and a half years after the removal of silicone oil. (a) An ultrawidefield fundus photograph showing retinal reattachment with retinal scars after photocoagulation. An outer retinal break is observed (arrow). The pale area indicates the location of the inner retinectomy. The visual acuity in the left eye was 20/60. The lines indicate the location of OCT scans presented in (b) and (c). (b) A horizontal optical coherence tomography (OCT) image showing the attached macula and thinning of the temporal retina. (c) A vertical OCT image showing the attached macula and a scar formation at the inferior macula.

**Figure 5:**
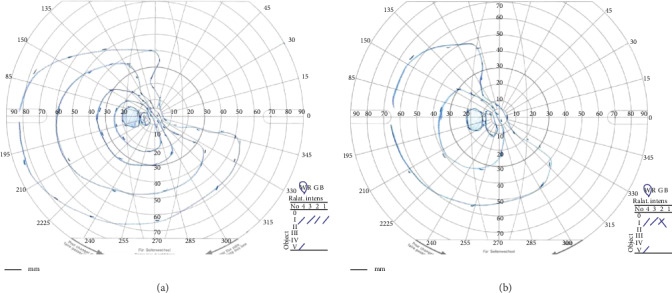
The visual field of the left eye before and after surgeries. (a) A preoperative Goldmann perimetry test before the initial surgery showing the defect of the superior nasal visual field corresponding to the area with branch retinal vein occlusion. (b) A postoperative Goldmann perimetry test at three months after the silicone removal showing narrowing of the nasal visual field slightly, but the majority of the visual field was retained.

A 61-year-old Japanese woman presented with declining vision in her left eye. She was diagnosed with BRVO in her left eye and underwent photocoagulation of the retinal periphery five years before this event. She had no history of systemic disease except for hypertension, controlled by medications. She developed retinal schisis extending to the macula and was referred to Chiba University Hospital for treatment. Her visual acuity was 20/20 in the right eye and 20/50 in the left eye at the initial presentation. The intraocular pressure was 12 mmHg in the right eye and 11 mmHg in the left eye. The axial length was 21.53 and 21.75 mm in the right and left eyes, respectively. The retinal schisis was observed at the inferior temporal retina with white vessels. Optical coherence tomography (OCT) showed macular traction and schisis. The posterior vitreous detachment was incomplete. The fluorescein angiogram showed retinal neovascularization in the nonperfused retina (Figure 1). She underwent phacovitrectomy with implantation of intraocular lens. During the surgery, the epiretinal membrane and internal limiting membrane were removed from the macula using Brilliant Blue G. The posterior hyaloid was separated except for the inferior temporal area where the vitreous is firmly attached to the retina. After the surgery, the decrease in the schisis cavity was observed with stable visual acuity between 20/50 and 20/40 (Figure 2). Two and a half years after the initial surgery, she presented with macula-involving bullous retinal detachment (Figure 3). Her visual acuity decreased to counting fingers. The outer retinal break was suspected posterior to the equator in the retina with BRVO. She underwent PPV with inner retinectomy at the BRVO area to reduce vitreous traction because the hyaloid was impossible to be separated from retina. The retinectomy encompassed the inferotemporal peripheral retina and extended posteriorly into the macula, almost to the fovea (Figure 4); note that the inferotemporal arcade was truncated by the retinectomy. Subretinal fluid was drained through the outer retinal break, and the retina was attached with silicone oil tamponade. The retina remained attached after the removal of silicone oil and the addition of the 7 mm width encircling buckle to support the peripheral retina. Her visual acuity was 20/60, and intraocular pressure was 10 mmHg at one and a half years after the last surgery (Figure 4). The defect in her visual field slightly enlarged after the inner retinectomy (Figure 5).

## 3. Discussion

RRD is a rare complication of BRVO [6, 9, 10]. The detachment can occur due to retinal breaks secondary to vitreous traction in the vicinity of retinal neovascularization [11–13]. In our case, the chronic BRVO developed retinal schisis, and the schisis gradually extended into the macula. We treated this patient with phacovitrectomy and removed the epiretinal membrane and internal limiting membrane to reduce macular traction. The schisis improved; however, retinal detachment developed later. We speculated that the residual vitreous caused traction and outer retinal break in the area of schisis. We had already known that the visual field was lost within the schisis and performed inner retinectomy to eliminate the vitreous traction. Since scleral buckling could support the vitreous base but not posterior traction, we suggest that the inner retinectomy plays a more important role in the reattachment of the retina in this case. The usefulness of inner retinectomy has been reported in the eyes with congenital schisis [14] and schisis associated with retinopathy of prematurity [15]. In our BRVO case, the procedure was effective, resulting in the reattachment of the retina with favorable visual recovery and minimal loss of the visual field. We suggest that the inner retinotomy is tolerable when the visual function disappeared in the area of schisis with persistent vitreoretinal traction. The efficacy of PPV for the treatment of RRD in BRVO cases has been reported [16], with better visual outcome in cases that lack vitreous adhesion [17]. Eyes with tractional tear more frequently presented macula-off RRD which required reoperation more often. Our case also showed firm adhesion of the vitreous, and late development of RRD occurred. It seems essential to follow cases where vitreous traction was not completely released.

In conclusion, we experienced a BRVO case with macula-involving retinal schisis treated with PPV. After the initial surgery, RRD developed and was successfully treated with PPV with inner retinectomy extending into the temporal macula. Retinal schisis is a rare complication of BRVO and may contribute to the risk of RRD via vitreoretinal traction.

## Data Availability

No data is available.
